# Nursing Student Experiences of Caring for Burned Patient: From Fearfulness to Normalization

**DOI:** 10.17533/udea.iee.v38n1e09

**Published:** 2020-02-26

**Authors:** Fahimeh Alsadat Hosseini, Marzieh Momennasab

**Affiliations:** 1 Nurse, Ph.D. Department of Medical-Surgical Nursing, Faculty of Nursing and Midwifery, Shiraz University of Medical Sciences, Shiraz, Iran. Email: fhosseini23@yahoo.com Shiraz University of Medical Sciences Shiraz Iran fhosseini23@yahoo.com; 2 Ph.D. of Nursing. Associate Professor, Department of Medical-Surgical Nursing, Faculty of Nursing and Midwifery, Shiraz University of Medical Sciences, Shiraz, Iran. Email: momennasab@sums.ac.ir. Corresponding author Shiraz University of Medical Sciences Shiraz Iran momennasab@sums.ac.ir

**Keywords:** qualitative research, attitude, students, nursing, nursing care, burn units, education, nursing., investigación cualitativa, actitud, estudiantes de enfermería, atención de enfermería, unidades de quemados, educación en enfermería., pesquisa qualitativa, atitude, estudantes de enfermagem, cuidados de enfermagem, unidades de queimados, educação em enfermagem.

## Abstract

**Objective.:**

To describe the care experiences of students in burn units.

**Methods.:**

Qualitative research of the phenomenological descriptive type which was conducted with the participation of eight senior nursing students in Shiraz College of Nursing and Midwifery, Iran. The method used for gathering data about Student experiences in Care Services for Burn Cases was the individual semi-structured interview. The Colaizzi method was used for analysing and interpreting the data.

**Results.:**

Three main themes emerged: *the attractive but stressful experience*, *trying to adjust* and *metamorphosis in attitude*. Taking care of burned patients led to metamorphosis and adaptation to the requirements of burn care due to the students’ improved attitudes, awareness and potentials. This finally turned the stressful nature of taking care of a burn patient into an attractive experience for them.

**Conclusion.:**

Students with little clinical experience of stressful working situations in burn units faced different challenges. Due to the specific nature of taking care of burned patients, the clinical experiences of nursing students who offer these services are unique.

## Resumen

**Objetivo.:**

Describir las experiencias de atención de los estudiantes en las unidades de quemados.

**Métodos.:**

Investigación cualitativa del tipo descriptivo fenomenológico que se realizó con la participación de ocho estudiantes senior de enfermería en el *Shiraz College of Nursing and Midwifery* en Irán. El método utilizado para reunir datos sobre las experiencias de los estudiantes en los servicios de atención de pacientes quemados fue la entrevista individual semiestructurada. El método Colaizzi se utilizó para analizar e interpretar los datos.

**Resultados.:**

Emergieron tres temas principales: *la experiencia atractiva pero estresante*, *tratando de adaptarse,* y *la metamorfosis en actitud*. El cuidado a los pacientes quemados condujo a la metamorfosis y a la adaptación a los requerimientos del cuidado de las quemaduras, debido a que los estudiantes mejoraron las actitudes, mostraron mayor conciencia y desarrollaron potencialidades. Esto finalmente convirtió la naturaleza estresante de cuidar a un paciente quemado en una experiencia atractiva para ellos.

**Conclusión.:**

Los estudiantes con poca experiencia clínica de situaciones laborales estresantes pueden enfrentar diversos desafíos en las unidades de quemados. Debido a la naturaleza específica del cuidado de este tipo de pacientes, las experiencias clínicas son únicas para los estudiantes de enfermería.

## Resumo

**Objetivo.:**

Descreva as experiências de atenção do aluno em unidades de queimados.

**Métodos.:**

Pesquisa qualitativa, do tipo descritivo fenomenológico, realizada com a participação de oito estudantes seniores de enfermagem da Faculdade de Enfermagem e Obstetrícia Shiraz, no Irã. O método utilizado para coletar dados sobre as experiências dos estudantes nos serviços de queimados foi a entrevista individual semiestruturada. O método Colaizzi foi utilizado para analisar e interpretar os dados.

**Resultados.:**

Surgiram três temas principais: a experiência atraente, porém estressante, tentando se adaptar e a metamorfose na atitude. Cuidar de pacientes queimados levou à metamorfose e adaptação às exigências do cuidado com queimaduras, porque os alunos melhoraram atitudes, mostraram maior consciência e desenvolveram potencialidades. Isso finalmente transformou a natureza estressante de cuidar de um paciente queimado em uma experiência atraente para eles.

**Conclusão.:**

Alunos com pouca experiência clínica em situações estressantes de trabalho podem enfrentar vários desafios em unidades de queima. Devido à natureza específica do atendimento a esses pacientes, as experiências clínicas são únicas para os estudantes de enfermagem.

## Introduction

Nurses, as key members of burn care teams, are responsible for designing a care plan based on patient needs, which change during the treatment process and the different phases of convalescence.(1) However, providing care services in burn units is challenging; it affects the nurses’ perceptions and creates unforgettable experiences that are not recognizable in other units.(2) The nurses’ first encounter with burned patients and burn units in Iran happens during their training period. The quality of the nursing education is closely related to the quality of the students’ clinical experiences. If students experience any pressure in educational settings, their learning may suffer.(3) Despite the incontrovertible role of clinical experiences in the acquisition of knowledge and skill, many nursing students consider them as stress-causing factors and clinical settings, like burn units, are stressful work settings.(4) 

Although there have been some efforts to describe the needs and experiences of nursing students, these studies mainly focus on general aspects, like problems in clinical teaching, the characteristics of an efficient clinical instructor, teaching strategies or the setting influences, in the form of quantitative research.(5-7) There are not enough researches specifically done on nursing student experiences in providing care services in the stressful burn unit. Since clinical education is a primary part of the nursing educational system, curriculum designers must make an attempt to provide suitable conditions for students so that they can acquire necessary skills peacefully, particularly with regard to burn care challenges for care service providers and their influences on students’ educational, psychological, and professional dimensions.(2) Therefore, investigating and evaluating clinical experiences of nursing students in providing care services for burned patients has a significant role in improving the quality of nursing education and care of burn injuries and conducting qualitative research based on nursing student experiences ; also, perceptions of taking care of patients can help the instructors and curriculum designers to offer efficient opportunities for learning. Hence, the present study was conducted with the purpose of describing the nursing students’ experiences in the burn unit.

## Methods

Design. Since one of the most important ways of identifying the students’ clinical experiences is using the phenomenological method,(8) and that burns caring experiences and their dimensions in students are not investigated yet, then to create a thorough and deep perception of nursing student experiences when facing burned patients and deal with taking care of them, descriptive phenomenological method was used. 

Participants and setting. This phenomenological study was performed on nursing students of Shiraz College of Nursing and Midwifery as a subset of Shiraz University of Medical Sciences in Iran. The inclusion criteria were being a senior nursing student who has done an internship in burn unit, unemployment in the clinical setting, being willing to participate in the present work, and being able to describe their experiences. Sampling was done purposefully. In Iran’s common nursing educational curriculum, the burn unit internship, course lasts for 12 days, during which students spend one week in the burn unit and another week in the burn emergency unit taking care of burned patients. In the internship course, each instructor teaches a group of between seven and eight students. In the present study, sampling was done purposefully among students with rich and copious insights. Therefore, in this study, eight undergraduate nursing students who had passed between one and four weeks of their burn unit internship course in different groups were selected purposefully. Each participant declared their consent and willingness to participate in this study. The setting was Shiraz College of Nursing and Midwifery. 

Data collection. The data were collected from January to August 2018. The data collection method used for this study was that of interviewing every participant individually that was done by the main researcher; for this purpose, eight in-depth and semi-structured interviews were performed. Interviews were held in a private place and arranged according to the participant’s will. In the beginning, the interviews were less structured and the interviewer started with one or two main questions and continued the interview based on the interviewee’s responses. The beginning of the interview was focused on the following main questions: ‘What is burn care in your view?’, ‘What was your feelings and thoughts during the internship?’and, ‘What was the experience of learning of care for burned patients?’ The questions that followed were based on the interviewees’ responses to these questions. Where necessary, follow-up questions were used to increase the clarity of information. The approximate duration of the interviews was about 45 to 60 minutes. The interviews were recorded after obtaining permission from the students. Each interview was listened to several times immediately after it was over, and was subsequently transcribed. They were also analysed after being performed; accordingly, the next interview was then planned. These interviews continued until data saturation was reached. Saturation is achieved when a new category does not appear and the categories reach saturation in terms of their features and dimensions.(8) 

Data analysis. The phenomenological analysis starts with bracketing the researcher’s subjectivity which refers to clarifying preconception throughout the study. This process is described as Epoché,(9) and it refers to setting aside the researcher’s prejudgments and predispositions towards the phenomenon. For this purpose, the researchers wrote a complete description of the phenomenon and before starting the data analysis, they read their subjectivity statement, including the description of their own experience with the phenomena. To analyse the data, Colaizzi's(8) seven stage method of data analysis was used, which consisted of reading participant descriptions, referring to protocols and extracting important phrases, forming meaning and concept for each phrase, categorizing the concepts based on topics, compiling the findings into one comprehensive description and finally returning the results to the participants. All stages of the data analysis were reviewed by the researchers’ team.

Study rigour. To ensure trustworthiness of the study, Guba and Lincoln’s(10) criteria were utilized. Dedicating sufficient time to data gathering, revision of the interview transcriptions by the participants, getting help by means of feedback from two colleagues familiar with qualitative research methods and from two experienced instructors from the undergraduate teaching level, plus the use of a negative case analysis, revision by external members and supervisors, a teamwork attitude, and researcher experience as an instructor in the field of nursing student training in the burn units and her trend to improve students' clinical experiences and learning besides improving the quality of care for burn patients, all contributed to the enhanced credibility of the findings. Furthermore, independent analyses conducted by each member of the research team and team analyses improved the credibility of the research. Also, detailed descriptions of the study were used to validate dependability and conformability. In addition, including precise instances and quotes from the participants was used for assuring the transferability of the findings.

Ethical considerations. After receiving permission from the ethics committee located in the research centre of Shiraz University of Medical Sciences (No. 14432), the individuals meeting the intended criteria to enter the study were identified, and after they had been presented with oral and written details, informed consent was obtained. The participants were assured about the confidentiality of the interviews and the presentation their interview data anonymously. The researcher gave the participants the chance of revoking their cooperation by providing their phone number and e-mail address in any phase of the project. They were also reassured that their withdrawal would not have any negative consequences for their education.

## Results 

The participants of this study consisted of eight senior nursing students. The majority of them were female (62.5%) and single (75%). The mean (with standard deviation) participant age was 24. The demographic characteristics of the participants are presented in Table 1.

**Table 1:** The demographic characteristics of the participants

Participants	Gender	Age (year)	Marital status	Student working experience in burn unit
P1	Female	22	Single	No
P2	Female	23	Single	No
P3	Female	22	Single	No
P4	Female	24	Single	No
P5	Female	24	Married	No
P6	Male	23	Single	No
P7	Male	32	Married	No
P8	Male	22	Single	No

The findings were organized in three main themes of “the attractive but stressful experience”, “trying to adjust”, and “metamorphosis in attitude”, together with 9 subthemes. In Table 2, all the main themes and subthemes are presented.

**Table 2:** The extracted themes from the participants’ experiences of giving care services to burned patients

Main themes	Subthemes
The attractive but stressful experience	The stress feeling The tendency to learn new and specialized skills
Trying to adjust	Positivity Empathizing with patients Considering professional principles Getting involved in taking care of patients under the instructor’s supervision
Metamorphosis in attitude	Fearfulness and negative attitudes before becoming involving in the care Gradual change of attitude Normalization

The attractive but stressful experience

The participants not only did not have a good feeling about giving care services to burned patients and were not willing to take care of them, but also believed that as a nurse they need to know how to take care of burned patients and acquire the requisite skills. In addition, the students experienced activities which were completely new to them and they did not have the opportunity of performing similar kinds of care services in the other units. The main theme of *the attractive but stressful experience* consists of the two subthemes *the stress feeling*, and *The tendency to learn new and specialized skills.*

The stress feeling. Many of the participants considered giving care services to burned patients a tough and stressful job. *When I enter the burn unit and I want to face a burned patient, I feel really scared let alone taking care of him, it is really stressful.* (P. 2) The reasons they mentioned were the tough nature of taking care of burned patients and the psychological effects that the patients’ conditions had on them. *I was really stressed out of taking care of burned patients because they are already in a bad condition so when we try to provide care services, such as a burning shower and debridement for them, they experience an acute pain*. (P. 3). Individual features of the participants and the stressful setting of the burn unit were also important factors influencing their stress feelings: *It’s really difficult for me to debride a burned patient, because he/she is in a lot of pain and I don’t like to see them suffering…I’m an emotional person, I feel stress when I watch burn wounds*. (P. 8). 

The tendency to learn new and specialized skills. Despite the tough nature of providing care services for burned patients and the psychological impact it has on nurses, the participants believed that this type of care service was somehow attractive to them. What made the internship in the burn unit attractive was the novelty of the learning, skills and experiences the interns acquired: *Generally, seeing it for the first time was attractive, it’s tough but attractive*. (P. 7) *It was a completely different experience…. very different, it was new, therefore, I tended to learn and do burn care.* (P. 2) The individual characteristics of the students are definitely influential in this regard: *Well, I think it depends on the student. For instance, I have a friend who says when I see that, I feel nausea, but I’m not like that. I like to provide care services for burned patients, dress them or do things like that, because it’s so interesting for me*. (P. 1).

Trying to adjust. Since taking care of burned patients was difficult and stressful for students in the beginning, many of the participants mentioned cases that expressed their effort to reach a level of adjustment when talking about their experiences of providing care services for burned patients. The subthemes of *positivity* toward providing care services, empathizing with patients, *considering professional principles,* and getting involved in taking care of patients under the instructor’s supervision all show their effort to reach a state of adjustment.

Positivity. One strategy that students use to adapt to the stress of working in the burn unit is thinking positively and looking for positive aspects of the experience. *However, we learn a couple of care services that can be applicable at home or somewhere else.* (P. 8) the students felt the significance of learning how to take care of burned patients, finding it useful in their personal life: *Unlike many illnesses and physical problems, everyone, may have experienced himself or his relatives, burn events, in a low or high levels, therefore, it is necessary to know the care services for burned patients.* (P. 7) Also, the participants perceived that teaching patients was so enjoyable: *Well, the act of teaching the patient itself is very enjoyable for me, because I know they don’t know some points that are very basic and then I explain these to them, I really like this job*. (P. 1)

Empathizing with patients. Another strategy that students use to adjust is being empathetic with patients. The horrible appearance of the patients and other physical, psychological, and financial problems imposed on them all make the students feel empathic and perform care services empathetically; hence, they do their best to provide care services and instruction for these patients regardless of their own personal will: *I said to myself [that] the most important thing is just helping this patient. I have to disregard some tough scenes to be able to provide the necessary services. Anyway, we are nurses and we have to be ready for everything. I tried to think less about trivial matters.* (P. 3) *As a nurse, I tried to pay more attention and sympathize with burned patients due to their physical and psychological conditions.* (P. 4)

Considering professional principles. The students participating in the present study felt the need for theoretical and practical learning about burns. This is perhaps because of the applied nature of burn teaching in their daily life and also their professional performance. Despite the tough nature of the internship in the burn unit, the participants found the internship course essential in improving their skills and adding to their knowledge. It was also good for meeting the expectations of others by whom they wish to be seen as qualified to provide health services: *For a nurse, being experienced in all units is necessary, and [the] burn unit is not an exception; then we go to work…the community expects us to have the related information about the healthcare services we provide for burned patents.* (P. 8) *I think it’s good, it was a useful internship. I really learnt a lot in the burn unit; [the lessons] were all applicable.* (P. 2) Also, due to the professional responsibility that students felt for these patients and also considering their special condition, students tried to overcome their negative feelings to provide care services. *When I think about the main goal of nursing, I can overcome my fears and do my best to take care of the patients.* (P. 3). *It is not right that because of unpleasant appearance of burned patients, we neglect our care duties for them*. (P. 4)

Getting involved in taking care of patients under the instructor’s supervision. The students felt the necessity of performing caring tasks during the internship course and felt their professional responsibilities to the patients. The students took care of burned patients under their instructor’s supervision and support, regardless of their internal feelings about the process. The instructor’s peace, dominance, and involvement in providing care service tasks made the students see her as an idol. This helped them to adjust and become more involved in their jobs. *Our instructor is really good…very much. She faces them very indifferently so as to encourage us to act like her in a way that leads us all to peacefulness. She simply gets into action and moves us into the task. When we are working with her, she’s a great source of motivation*. (P. 2)

Metamorphosis in attitude. Before starting the burn unit internship, there was a negative view about taking care of burned patients among students, but as the time passed, and in accordance with the increase in their care experiences, a change occurred in their feelings and attitudes. Being in a care-giving situation gradually changed the participants’ attitudes and led to a change in their feelings, and a decrease in the fear and hatred they had towards taking care of burned patients. Time also helped to normalize the job for them. This main theme included three subthemes: *Fearfulness and negative attitudes before becoming involving in the care, gradual change of attitude*, and *normalization*.

Fearfulness and negative attitudes before becoming involving in the care. There is a negative mentality about taking care of burned patients in many students before they begin their internship in the burn unit. One important factor for this fear is the nature of burn injuries; another can be the senior students’ stressful experiences in the burn unit. Because of this negative attitude, many of the participants were very fearful about taking care of burned patients. Before the beginning of clinical burn course, I was very scared. I thought it would be too stressful to see burned patients and take care of them. (P. 3) Other guys who came for [the] internship said that it’s tough. (P. 1) Well... burned patients have [a] horrible appearance, we have this fear unconsciously. (P. 5)

Gradual change of attitude. After students were settled into giving care services that accounted for the burned patients’ situations and once they had experienced this kind of caring, they experienced both an increase in their knowledge and skills in this regard and a gradual decrease in their fear; therefore, their attitude improved comprehensively. As I took care of various burned patients, my attitude about burn care changed. (Male, aged 22) Then, I felt it got easier and it wasn’t that much hard, maybe because I was in that setting. (P. 2) when I got involved in caring of patients in this clinical course, over time, my views about the course and burn nursing changed dramatically. (P. 1)

Normalization. After spending a certain amount of time in giving care services in the burn unit, the students’ fear and stress diminished, their attitudes changed, their knowledge and skills improved, and because of their persistence in practising the job, the students gained a level of normalization with respect to their feelings about giving care services to burned patients. It gets normal, I mean when you see them, you feel a little less emotional, but you still have that bad feeling…but that’s no problem, because it gets normal. Anyway, when you see a burned patient several times, you get used to it. (P. 6)

The more, time passed from the burn care, the less I got stressful than before. (P. 8)

## Discussion

Owing to the particular condition of burned patients, taking care of them is considered a unique and different experience for nursing students. Although it is clear that this kind of experience can be effective in promoting learning and designing the educational curriculum for students, it is also necessary to study their experiences in this regard closely. In studying the experiences of nursing students in burn care units, the findings are presented in the form of three main themes and 9 subthemes: *the attractive but stressful experience* (Including the subthemes of *the stress feeling*, and *the tendency to learn new and specialized skills)*, *trying to adjust (*Including the subthemes of positivity, empathizing with patients, considering professional principles, and getting involved in taking care of patients under the instructor’s supervision*)* and *metamorphosis in attitude (*Including the subthemes of fearfulness and negative attitudes before becoming involving in the care, gradual change of attitude, and normalization*)*. Taking care of burned patients led to metamorphosis and adaptation to the requirements of burn care due to the students’ improved attitudes, awareness and potentials. This finally turned the stressful nature of taking care of a burned patient into an attractive experience for them.

One of the main themes identified according to the participants’ descriptions of burn care was that it was *the attractive but stressful experience*. The participants of the current study experienced stress feelings in taking care of burned patients; this feeling was affected by individual and environmental factors, and also by the tough nature of burn care. Nevertheless, they were aware of the significance of learning this kind of caring. On the other hand, not having any similar experience worked as an important motivating factor that made taking care of burned patients attractive to them, even as it was a stressful job. Both the act of taking care of patients with severe burn injuries and the experience of the burn unit conditions themselves are considered seriously stressful and emotional factors. Bayuo(2) believes that nursing in the burn unit is a serious challenge because of the physical and emotional demands imposed on nurses by patients. Nursing students, as well, face various emotional and cognitive challenges as soon as they enter the burn units. They even have negative presuppositions before entering the unit, because of the experiences that their peers have talked about which can have a negative impact on the learning of related topics.(11) Nonetheless, the participants of the present study described this unpleasant experience as being attractive. Bayuo,(2) in their study, described this experience as one filled with distress and unpleasant feelings. Likewise, according to the results of another study, taking care of burned patients, because of the participants’ need and their demands in both individual and professional dimensions, was mentioned as an attractive, particular field.(12) It seems that encountering new cases as well as comprehending the necessity of learning how to take care of burned patients during the burn unit internship with the purpose of meeting professional expectations made this internship course attractive despite the stress feelings and challenges of taking care of these patients. In the participants’ views, individual traits and emotions were the influencing factors in this experience. However, in order to increase the tendency of students to take care of burned patients and improve their learning, there is a need to reduce students' stress by removing their concerns and also preparing them for pre-internship. In the study by Kornhaber,(13) it was shown that emotional strength and feeling detachment are basic elements in facilitating the process of accepting the patient’s condition and following the procedures of giving care services to burned patients. 

Another theme extracted from the student interviews with respect to burn patient care was *trying to adjust*. The experiences of this study’s participants revealed their effort to utilize different adaptive strategies to reach an adjustment level in taking care of burned patients. Clinical settings, like burn units, are stressful work settings.(4) The Stress during this course can lead to several negative outcomes, such as poor academic performance, (3) increased burnout levels,(14) and reduced personal well-being.(15,16) All these factors are crucial to the achievement of the goal of training, which is to prepare competent nurses.(17) Because nursing students are not able to stay away these stressors in clinical setting, it is essential for students to cope with them.(18) With regard to the significance of learning burn care, and considering their professional commitments on one hand, and also empathizing with patients and comprehending their needs on the other hand, students tried to take care of these patients under their instructor’s supervision, thus coming to a positive attitude about the care they provided. This has been pointed out in other studies as well.(12,19) Consistent with the findings of the present study, and according to other research, nurses working in burn units benefit from different strategies for mitigating their stress and dealing with the challenges they encounter in taking care of burned patients, namely maintaining a positive attitude and getting involved in the care process,^(20, 21)^ being a pragmatic and committed nurse in the burn unit,(13) putting himself/herself in the place of patient and their family, showing empathy and compassion,(13,21) and supporting the leadership.(22,23) It seems that adaptation to the stress management challenges of burned patients is a prerequisite for the basic learning of burn care and participation in the care process of these patients. In this regard, step-by-step support of students prior to and during different phases of the internship, in addition to exploiting various adjustment sources like personnel cooperation, creating a supportive environment and counselling, can have a significant role in helping the students to pass these adaptive phases and reach the desired compatibility. 

The third extracted theme from the findings of this study was *metamorphosis in attitude*. Before they started their internship and burn care course, students had a kind of fear about providing care services in the burn unit because of what they had heard from other students; yet, by getting involved in care services in the burn unit, their attitude changed considerably and they came to a sense of normalization. In the study conducted by Kornhaber,(13) all the participants experienced apprehension in taking care of burned patients. Apparently, some part of the students’ first apprehension was due to their partial knowledge of burn care and of what they would be expected to do in the burn units. Therefore, after experiencing real care situations and gaining experience and skill in taking care of burned patients under their instructor’s supervision, and once they could visualize a realistic picture of the conditions in the burn unit, their attitudes improved and their fears faded away. It is probable that the students’ experiences and reflections increased over the course of spending time in burn units and, in practice, their attitudes also improved. The emotional reactions they showed in this field decreased noticeably. It seems that taking short-term measures in order to familiarize students with different aspects of burn care and eliminating ambiguity concerning intern duties before starting the internship course can comprehensively reduce the levels of apprehension and stress, and ultimately lead to sufficient preparation to ensure a positive experience learning burn care for students. 

Finally, it should be mentioned that nursing students, despite their preliminary noticeable fears in providing care services for burned patients, knowing that by necessity they will encounter novel care situations and desiring to attain new knowledge in this discipline, try to adjust to the circumstances of the burn unit, which ultimately results in a metamorphosis as their knowledge increases and their attitudes change. They attempt to make this adjustment notwithstanding the fact that it is a stressful experience; Nursing students are trying to care for burned patients under training and supervision of their clinical instructors. They take whatever pleasure there is to be had in the process of learning new requirements. Endeavouring to create a supportive environment before and during the internship course and encouraging positive attitudes in students while meeting their individual needs can be effective in decreasing the students’ stress feelings and improving their learning.

The strength of this study is the presentation of a comprehensive drawing of nursing students' experiences in a stressful clinical setting of burn using a phenomenological qualitative method. This can lead to providing strategies to improve this experience and learning of the students' and patients’ care in the burn units. Choosing participants from a teaching centre was one of the limitations of this study, a practice which led to less variety in the sample. Data gathering for this study was also performed through interviewing every participant individually, while using other data gathering methods, like observation, could lead to richer results for the qualitative research aspect. Conducting qualitative research on the students in other universities and burn centres as well as utilizing other methods for doing qualitative research could increase the credibility of the findings and help make them generalizable.

## Conclusion.

The findings of this phenomenological study showed that the real experiences of nursing students in taking care of burned patients were unique because of the traumatic nature of burn injuries in terms of both physical and emotional aspects. Based on this study, students, despite their preliminary apprehensions, tried to adapt to the stresses and challenges of providing burn care. In the course of the training path, improving their attitudes and increasing their knowledge and professional abilities resulted in a metamorphosis and an adjustment to the process of taking care of burned patients. Consequently, in spite of the stressful nature of the burn unit experience, it became more attractive for students. Educational and nursing officials and curriculum designers need to investigate the findings of this study and create a suitable educational setting with the purpose of helping the students benefit from this internship course, students’ better learning in this internship course, improving the students’ caring experiences and increasing the quality of care services provided for burned patients. In addition, in this regard, they must support the students emotionally, psychologically, and educationally before and during their internship course.
